# Idiopathic venous thromboembolism and thrombophilia

**Published:** 2011-02-25

**Authors:** C Sinescu, M Hostiuc, D Bartos

**Affiliations:** * ‘Bagdasar Arseni’ Clinical Emergency Hospital, BucharestRomania; ** Bucharest Clinical Emergency Hospital, BucharestRomania

**Keywords:** thromboembolism, hypercoagulability, antiphospholipid syndrome, recurrence

## Abstract

During the past decade idiopathic venous thromboembolism has become a separate entity, a chronic illness which has required prolonged anticoagulation and other prevention strategies to avoid recurrences. This article reviews recent developments regarding unprovoked venous thromboembolism and its relation with thrombophilia. In the beginning, the latest definition of idiopathic venous thromboembolism is presented. The article continues with statistics about thrombophilia, related venous thromboembolism, and a classification of major thrombophilic factors according to their intrinsic risk of thrombosis and of thrombotic recurrences. Great interest is given to the predictors of recurrence and the importance of prolonged anticoagulation is underlined. The antiphospholipid antibody syndrome, the most common acquired thrombophilia, is presented separately. The revised diagnosis criteria are discussed. Some characteristics of the antiphospholipid syndrome are worth presenting: the risk of both venous and arterial thrombosis, the high risk of thrombotic recurrence and the diversity of antiphospholipid antibodies.

Patients experiencing idiopathic venous thromboembolic event have a great risk of recurrence, and highly benefit from long time anticoagulation. Natural coagulation inhibitors deficiencies, homozygous factor V Leiden and prothrombin G20210A and the antiphospholipid syndrome, increase the risk of first venous thrombosis and their recurrences and require adequate prevention.

Abbreviations: VTE–venous thromboembolism, HRT–hormone replacement therapy, AVK–antivitamin K, FVL–factor V Leiden, PT G20210A–prothrombin G20210A, TAFI–thrombin activatable fibrinolysis inhibitor, PAI–1–plasminogen activator inhibitor 1, T–PA–tissue plasminogen activator, APS–antiphospholipid syndrome, LA–lupus anticoagulant, Abeta2GP1–anti beta2 glycoprotein 1.

## Background

Even if it is a common disease, venous thromboembolism may sometimes be challenging. In case of a patient with deep venous thrombosis or pulmonary embolism many questions arise: which was the cause, which is the most appropriate treatment, how long the treatment should be or how can we prevent a thrombotic recurrence. Since 1856 when Rudolf Virchow first described the three factors involved in the thrombotic process–hypercoagulability, hemodynamic changes (stasis, turbulence), endothelial injury/dysfunction–a lot of progress has been made in understanding thrombosis.[1] Since 1965 when Egeberg described  for the first time a case of familial thrombosis determined by antithrombin deficiency, numerous mutations causing hypercoagulability have been discovered. [1]

### Idiopathic Venous Thromboembolism

Unprovoked (idiopathic) VTE definition differs from study to study, the most recent being published in the French consensus guideline in 2009 [1], where idiopathic VTE is defined as being the VTE which occurs in the absence of triggering circumstances classified as it follows:

- Major triggering circumstances: Plaster cast immobilization and /or fracture of a lower limb or surgery under general anesthesia lasting for more than 30 minutes or bed rest for more than three days, occurring in three previous months or active cancer in the two preceding years.
- Moderate or minor triggering circumstances: Pregnancy or post–partum, oestroprogestative contraception or HRT in the year preceding the VTE, a journey lasting for more than 6 h. [1]

The incidence of unprovoked VTE varies between 25% and 50% as reported in different studies. [2] The studies demonstrated that in almost 50% of first VTE event, a thrombophilic factor could be identified. [3] Testing for thrombophilia is recommended, but there are controversies with respect to the way thrombophilia influences the anticoagulant treatment duration, the risk of recurrence, the benefice of testing the asymptomatic relatives. 

### Thrombophilic factors

Recently, a stratification of  major thrombophilic factors according to their risk of thrombosis has been published.[4] Based on this study the most thrombogenic factors are  deficiencies of natural coagulation inhibitors (antithrombin deficiency, protein C deficiency, protein S deficiency).[4]  Also, a high risk of thrombosis has been noticed in the case of antiphospholipid  syndrome and in the homozygous forms of factor V Leiden and prothrombin G20210A. Heterozygous forms of factor V Leiden and prothrombin G20210A and high levels of factor 8 are responsible only for a moderate increase of the risk of thrombosis. [4] Considering these, it becomes obvious that acquired risk factors for thrombosis have an important role and that, in many cases, venous thromboembolism occurs in the presence of both acquired and genetic thrombotic factors.

Testing for thrombophilia is recommended in:

- Unprovoked VTE  before 60 years old or VTE occurring in the absence of major triggering circumstances;
- Family history of thromboembolic disease;
- Thrombosis in unusual sites: cerebral veins, visceral veins thrombosis, upper extremities vein thrombosis etc.;
- Obstetric pathology like: recurrent miscarriages, stillbirths or dismature  newborns;
- Abnormal laboratory tests without apparent causes (prolonged aPTT);
- Skin necrosis after initiation of AVK treatment.[5]

Another problem is whether testing for thrombophilia, the asymptomatic relatives of patients with certain thrombophilic defects, is needed. The Belgian Society on Thrombosis guidelines recommends a well–balanced decision, considering the stress, anxiety and over protective attitudes generated by thrombophilia.[5] French consensus guidelines recommends to test the asymptomatic relatives when the index case suffers from antithrombin deficiency (except for the mutation of the heparin binding site, the ⅡbHBS variant), protein C and S deficiencies and also in the case of homozygous  or double heterozygous forms of factor V Leiden and PT G20210A. It seems to be useless to test the asymptomatic relatives of an index case with heterozygous form of FVL or PT G20210A, because the studies revealed that the risk of thrombotic events is not significantly higher than the risk of other persons with the same risk factors from the general population. That explains why family studies are not recommended in case of moderate thrombophilic factors with high prevalence in general population. Also, considering the costs, it is recommended to begin the screening of asymptomatic relatives with the mutation found in the index case. If the test is negative, the recommendation is to stop the screening, but if the test is positive, the screening should continue, as combined thrombophilia might coexist and increase the risk of thrombosis.

There are also problems regarding the moment of testing for thrombophilia in order to obtain a reliable result, especially in patients with a first VTE.

During the acute thrombotic episode, many factors may distort the results: factor's 8 levels may be higher than normal (acute phase reactant), protein C, S and antithrombin levels may be lower than normal. Also antithrombin levels may be under the inferior limit during the therapy with unfractionated heparin. Protein C and S levels (which are vitamin K dependent proteins) are reduced by vitamin K antagonists, explaining why testing for their deficiencies is not recommended while using anticoagulants.[6] According to the general recommendations, the most appropriate moment for thrombophilia screening is at the end of the anticoagulant treatment, usually 4 or 6 weeks later.[5,6] However, some tests could be performed in the acute episode: lupus anticoagulant and anticardiolipin antibodies (IgM and IgG), protein C, S and APCR in case of skin necrosis, antithrombin in case of thrombosis at unusual sites or at young age. [5] Genetic analysis for factor V Leiden and PT G20210 detection may be performed during the acute episode but considering their costs, it is preferable to wait and screen initially for APCR and for natural anticoagulants deficiencies.

Another problem regarding thrombophilia is if screening should be performed before starting oral contraception and hormone replacement therapy. With respect to this problem, the French consensus guideline sustains that the presence or absence of thrombophilia does not change the clinical attitude in the context of the prescription of an oral contraceptive to a patient with history of VTE, as taking oral oestroprogestative contraceptives or hormone replacement is contraindicated in women with previous VTE. However, there are no indications if screening for thrombophilia should be done before taking contraceptives in women without a history of VTE.

### Predictors of recurrence 

There are also controversies regarding the length of the anticoagulant treatment in patients with venous thromboembolism and thrombophilia. As the risk of thrombosis is higher than normal, it seems reasonable to administrate anticoagulants for a longer period of time in order to prevent recurrences. Many researchers focused on this problem, but, unfortunately, the results are not as they expected, because some studies failed due to the slow inclusion rate, other used different cut off values and their result could not be compared or had divergent conclusions, others were too small to have a powerful statistical significance or used different definitions for ‘unprovoked VTE’ or for ‘family history of VTE’. After analyzing the existing studies, the French group of specialists concluded that in the assessment of the risk of thrombosis recurrence the most important step is to determine whether the episode was provoked or not, the unprovoked (idiopathic) character of VTE being a strong indicator of recurrence, irrespective of any knowledge of possible thrombophilia. In a prospective cohort study of 570 patients with a first episode of VTE, Baglin et al demonstrated that at two years after ceasing the anticoagulant therapy, the cumulative recurrence rate was 11%. [7]. The statistical analyses revealed that the most important predictor of recurrence was the presence of clinical risk factors for thrombosis. The recurrence after a surgical related VTE was of 0%, and the highest recurrence rate was noticed in the unprovoked episodes of VTE (19.4%, p<0.001). Also, the results showed that in patients with unprovoked VTE or with a non–surgical related VTE, the recurrence rate did not differ between patients with or without thrombophilia.[7]. 

Another predictor of recurrence after a first VTE event is the age. It is very well–known that the incidence of VTE increases with age, and about 70% of patients presenting a first episode of VTE are over 60 years old.[1]. The rate of recurrence is higher when the first episode of VTE occurs before 60 years old, as studies proved. [1]. Also, the French guide underlines that in the age group over 70 years, the presence of thrombophilia does not seem to influence the risk of recurrence due to age, particularly with regard to factor V Leiden and FIIG20210A polymorphisms. [1]. Regarding the natural coagulation inhibitors deficiencies, conclusions are difficult to be drawn because of the lack of information, most of it coming from family studies and not from the general population. From the available data, it results that antithrombin deficiency, protein C deficiency and protein S deficiency have a recurrence risk of 40% at 5 years and of 55% at 10 years comparative to 11% at 5 years and 25% at 10 years for factor V Leiden, Prothrombin G20210A and high factor 8. [4]. In Brower et al study concerning 130 patients with natural anticoagulants deficiencies, with first VTE, the annual ratios of recurrence were of 8.4% for protein S deficiency, 6% for protein C deficiency and 10% for antithrombin deficiency, compared to an estimated recurrence rate of 1% in patients without a deficiency. [8].They concluded that patients with coagulation inhibitors deficiencies have a high absolute risk of recurrence and that this risk is increased after a first spontaneous event and when associated with other thrombophilic defects.[8]

Another problem is that inhibitors deficiencies are caused by different mutations, each of them determining a different risk of thrombosis, which explains the variety of phenotypes among people with the same anticoagulant deficiency. An edifying example is antithrombin deficiency, recognized as one of the most important risk factors for thrombophlia–induced thrombosis. Until now, more than 150 mutations of the antithrombin gene have been identified. Most of them are found in the heterozygous form. The homozygous cases are very rare (less than 20 reported) and are associated with severe and early onset of the thromboembolic disease (with both venous and arterial thrombosis) and often without a family history of thrombosis. The risk of thrombosis induced by the type Ⅱ deficiency of heparin binding site (ⅡbHBS variant) is very low, compared to other congenital deficiencies of antithrombin. It has also been noticed that only the mutations that in the heterozygous form induce a low or moderate thrombotic risk could be seen in a homozygous form, otherwise the homozygous status is lethal.

Trying to answer the question: ‘When is the best moment to stop oral anticoagulation treatment?’, Palereti et al suggested that  D–Dimer assay may be used in the appreciation of the duration of oral anticoagulant treatment and to optimize prevention of recurrent venous thromboembolism. They evaluated 599 patients with a previous VTE, who were repeatedly tested for D–Dimer levels after interrupting oral anticoagulation and were also screened for most frequent thrombophilic alterations. They concluded that normal D–Dimer levels measured one month after oral anticoagulant treatment withdrawal have a high negative predictive value (92.9%) for recurrence in patients with idiopathic VTE and in carriers of inherited thrombophilia. Increased D–Dimer levels were associated with a significantly higher hazard ratio for VTE recurrence. [9]

Another method, which seems to help the clinician establish the risk of recurrence, is examination of residual venous thrombosis on ultrasound. Patients with residual thrombosis have a higher risk of recurrence that those without remaining thrombi. 

However, until now, neither the D–Dimer levels nor the residual thrombosis are precise methods to predict which patients will experience a recurrence after the cessation of the anticoagulant treatment.

### Anticoagulant treatment in idiopathic VTE

S. Goldhaber underlines the importance of considering the idiopathic VTE as a chronic disease and insists on the benefits of indefinite duration of anticoagulant treatment, possibly for a lifetime. His affirmations are based on studies like PREVENT, ELATE and THRIVE. [10] The PREVENT (Prevention of Recurrent Venous Thromboembolism) trial demonstrated the advantages of low–intensity anticoagulation with warfarin (target INR of 1.5–2.0) in 508 patients with idiopathic VTE after six months of standard anticoagulation. The trial was interrupted after two years due to the obvious benefits noticed in the warfarin group compared to the placebo group. The ELATE (Extended Low–Intensity Anticoagulation for Thromboembolism) study of 738 patients with unprovoked VTE compared the benefits of low–intensity warfarin anticoagulation (target INR between 1.5 and 1.9) to standard anticoagulation (target INR of 2.0– 3.0). The patients were included after they had completed at least three months of conventional anticoagulation and were followed up for a medium of 2.4 years. The results showed a likelihood of recurrence of 2.8 higher in the low–intensity anticoagulation group than in the standard anticoagulation one. Another important finding was that the adverse effects of anticoagulation (hemorrhage) occurred with a similar frequency in the two groups. The THRIVE study demonstrated the benefits of using a thrombin direct inhibitor (ximelagatran) in decreasing the recurrent thrombotic events in 1233 patients with idiopathic VTE after six months of standard warfarin anticoagulation. Regarding the drug used in THRIVE trial–ximelagatran–it has to be mentioned that in 2006 its manufacturer retired the product from the market because of reports of hepatotoxicity during trials. However, other molecules from the same class such as dabigatran seem to have the same beneficial effects and less aggressive side effects.

Considering the predisposition for thrombosis in thrombophilic patients, one can argue that thrombophilia may also induce an increased risk of VTE recurrence while on warfarin treatment and that a high intensity of anticoagulation may be needed in order to prevent the recurrent events. A substudy of the ELATE trial demonstrated that single or multiple thrombophilic defects are not associated with a higher risk of VTE recurrence during anticoagulant treatment, with the possible exception of the antiphospholipid antibodies. [11] 

### Rare thrombophilic factors

Regarding the rare thrombophilic defects like dysfibrinogenemia, elevated TAFI, elevated PAI–1, t–PA deficiency, few data are available, mostly as case reports. They are not part of the screening analyses and due to their low prevalence, large studies are impossible to be realized, making a discussion about their influence on the recurrence risk rather difficult. 

High factor 9, high factor 11, high TAFI are not independent risk factors for venous thromboembolism, but become risk factors when associated with high factor 8 levels. [4]

It has been demonstrated that elevated factor 8, generally defined as above 90 percent, determines an increased risk of recurrent VTE.[1] In different studies, this risk is clearly related to the plasma concentration of factor 8 and the threshold used to define how a high factor 8 influences the risk of recurrence, and explains the variable results between different studies.

Hyperhomocysteinemia is also a well–known risk factor for both venous and arterial thrombosis. Severe hyperhomocysteinaemia has been noticed in homocystinuria, a pathology caused by enzymatic deficiencies, which affect the homocystein metabolism. Such patients often experience an arterial or venous thrombosis before the age of 30 years. As for factor 8, there are also problems with the definition of hyperhomocysteinaemia. Generally, it is considered when plasma concentration is higher than 95 percent. The Austrian Study on Recurrent Venous Thromboembolism found that hyperhomocysteinemia represents a risk factor for recurrent VTE [1], while others, like Leiden Trombophilia Study, disagree. [1]

### The Antiphospholipid Syndrome

A special attention must be given to the antiphospholipid syndrome. It represents an acquired thrombophilic disorder that can be seen as a distinct entity (primary antiphospholipid syndrome) or in association with systemic lupus erythematosus or other autoimmune diseases (secondary antiphospholipid syndrome). It increases both the risk of venous and arterial thrombosis. The diagnosis was established based on the Sapporo criteria. According to these, definite antiphospholipid syndrome is considered when at least one clinical criterion and one laboratory criterion are met.  The clinical criteria refer to the presence of (a) vascular thrombosis (venous, arterial or small vessels thrombosis confirmed by imagistic  procedures or hystopathologic exam) or to (b) pregnancy morbidity (premature neonates due to severe pre–eclampsia or severe placental insufficiency; fetal death at or beyond 10 weeks of gestation; three or more spontaneous abortions before the 10^th^ week of gestation). The laboratory criteria refer to the presence of anticardiolipin antibodies (Ig G or Ig M) at least in two determinations, at least six weeks apart and in the presence of lupus anticoagulant, and also, on two or more occasions with a minimum of six weeks distance between them. 

Recently, at a workshop in Sydney, Australia, the laboratory criteria have been modified by also including the antibodies against beta2– glycoprotein Ⅰ in the laboratory classification criteria in addition to lupus anticoagulant and anticardiolipin antibodies. In addition, the role of antiprothrombin antibodies and antiphosphatidylethanolamine antibodies was considered poorly investigated in order to include these antibodies in the diagnostic criteria. More recently, a group of Italian researchers, developed o study based on WAPS (warfarin in antiphospholipid syndrome study) in order to explore the relationship between single/multiple positive tests for LA and IgG/IgM for aCL, abeta2GPI, antiprothrombin antibodies, antiprotein S and antiannexin AV antibodies and thrombosis and obstetric complications.[12] They concluded that the introduction of abeta2GPI antibodies in the antiphospholipid syndrome diagnosis criteria was well justified and, went further, and proposed the replacement of aCL antibodies with abeta2GPI antibodies in the laboratory criteria, underlining the importance of IgG isotype of abeta2GPI antibodies. They also suggested that antiprothrombin and antiannexin AV antibodies could be considered as future candidates for the antiphospholipid syndrome criteria. [12]

After a first thrombotic episode (venous or arterial), the risk of recurrence is significant in patients with persistent antiphospholipid antibodies. Khamashata et al reported that 69% of the patients with APS included in their study suffered a total of 186 recurrences, with the median time between the initial thrombosis and the first recurrence of 12 months.  The highest rate of recurrence was noticed during the first six months after warfarin discontinuation. They concluded that the risk of thrombosis recurrence in patients with APS is high and long–term treatment is justified. They also recommended high–intensity anticoagulation for these patients. [13] Later, Finazzi et al demonstrated in WAPS study that high–intensity warfarin treatment was not superior to conventional anticoagulant treatment and it significantly increased the bleeding complications. [14]

Another problem is which is the best treatment in cases of arterial thrombosis (for example noncardioembolic stroke or transient ischemic attack) in patients with APS: antiplatelet agent or oral anticoagulant? Few randomized control trials are focusing on this problem, so, it should be treated according to the guidelines for stroke or myocardial infarction management. Moreover, secondary thromboprophylaxis measures such as hypertension control and lowering LDL–cholesterol levels are of great importance. [15] Noncerebral artery thrombosis such as renal thrombosis is recommended to be treated with moderate intensity oral anticoagulation indefinitely. [15]

## Conclusions

Idiopathic VTE requires a special attention from the clinician as he deals with a chronic patient who needs to be carefully investigated, periodically monitored, and treated for a long time, sometimes for a lifetime. A positive diagnosis of thrombophilia influences the therapy if endogenous anticoagulants deficiencies, homozygous forms of factor V Leiden and prothrombin G20210A or antiphospholipid syndrome are found.  Treatment should be individualized not only according to the type of thrombophilia but also to the patient’s comorbidities and additional risk factors.
